# Comparison of mechanisms involved in impaired vascular reactivity between high sucrose and high fat diets in rats

**DOI:** 10.1186/1743-7075-7-48

**Published:** 2010-06-04

**Authors:** Karen L Sweazea, Mateja Lekic, Benjimen R Walker

**Affiliations:** 1 College of Nursing and Health Innovation, Arizona State University, (1401 E Tyler Mall), Tempe, (85287-4501), USA; 2 Department of Cell Biology and Physiology, University of New Mexico Health Sciences Center, (1 University of New Mexico), Albuquerque, (87131), USA; 3 Department of Applied Science and Mathematics, Arizona State University, (7231 E Sonoran Arroyo Mall), Mesa, (85212), USA

## Abstract

**Background:**

To determine the effects of high sucrose diets on vascular reactivity. We hypothesized that similar to high fat diets (HFD), HSD feeding would lead to increased adiposity resulting in inflammation and oxidative stress-mediated impairment of vasodilation.

**Methods:**

Male Sprague-Dawley rats were fed control chow (Chow), HSD or HFD diets for 6 weeks. The role of inflammation and oxidative stress on impaired vasodilation were assessed in isolated mesenteric arterioles.

**Results:**

HSD and HFD induced increased adiposity, oxidative stress and inflammation. HFD rats developed fasting hyperglycemia. Both HSD and HFD rats developed impaired glucose tolerance and hyperleptinemia. Nitric oxide (NO)-mediated vasodilation was significantly attenuated in both HSD and HFD rats but was normalized by treatment with antioxidants or anti-inflammatory drugs. Endothelial NO synthase (eNOS) protein expression was not affected by diet. Sensitivity to NO was reduced since NOS inhibition attenuated vasodilation in Chow rats but did not further impair vasodilation in HSD or HFD rats. Likewise, responsiveness to a NO donor was attenuated in both experimental groups.

**Conclusions:**

Oxidative stress diminishes vasodilatory responsiveness in HSD and HFD rats through ROS-mediated scavenging of NO and decreased smooth muscle sensitivity to NO. Inflammation also plays a significant role in the impaired vasodilation.

## Background

Reactive oxygen species (ROS) have been implicated in the pathogenesis of many diseases including diabetes [1], cardiovascular disease, and ischemia reperfusion injury [2-5]. ROS can be produced through uncoupling of the electron transport chain or nitric oxide synthase as well as by NADPH oxidases [6-8]. Hyperglycemia as well as elevated plasma free fatty acids and triglycerides also contribute to increased ROS production in addition to initiating insulin resistance through down-regulation of insulin signaling pathway intermediates [9-11]

Endogenous antioxidant pathways protect from increases in ROS, but are often compromised in disease states. An important constitutive antioxidant pathway is the breakdown of superoxide anions (O_2_˙ˉ) by superoxide dismutase (SOD) to form hydrogen peroxide (H_2_O_2_), which is further converted by catalase or glutathione peroxidase to form water and oxygen [12-14].

Increases in the vascular levels of O_2_˙ˉ lead to endothelial dysfunction through scavenging of the endothelial vasodilator nitric oxide (NO) to form peroxynitrite (ONOO-) thereby decreasing the bioavailability of NO. In addition, diminished levels of the essential eNOS cofactor, tetrahydrobiopterin, results in uncoupling of eNOS and enhanced O_2_˙ˉ formation leading to further decreases in NO levels [6-8,11]

Elevated plasma glucose and triglyceride levels, which can lead to increases in oxidative stress [15], are thought to be involved in the pathogenesis of impaired vasodilation observed with high caloric intake and resultant increases in body mass. Increased adiposity is also associated with chronic inflammation [16,17] and may thus contribute to impaired vasodilation observed in overweight animals. The roles of inflammation and oxidative stress on responsiveness to the endothelium-dependent vasodilator acetylcholine (ACh) will be examined in the present study. The majority of research on the effects of high fat feeding on vascular function has been carried out using large conduit arteries such as the aorta from animal models of established obesity, including rats with high fat diet-induced obesity. Although these studies are informative in documenting the end effects of obesity, much less is known of the nature of vascular derangements in more modest settings of high caloric intake prior to overt obesity. Moreover, very little research has been conducted examining the effects of high sucrose feeding, in the absence of high fat intake, on vascular reactivity. In one recent study, inactive people who simultaneously ingested a candy bar and soft drink developed impaired endothelial function, as evidenced by lower flow-mediated vasodilation, compared to those who ingested the same meal but underwent a single bout of endurance exercise the day prior to blood flow measurements [18]. In studies of combined high fat and high sucrose feeding for 6 months, rats develop impaired aortic endothelial dependent vasorelaxation that is reversible following a low-fat complex carbohydrate diet [19]. Other studies have shown that feeding rats a combined high sucrose and moderately high fat diet for only 3 days similarly results in significantly impaired arterial vasorelaxation in the absence of weight gain [20]. Potential interactions between high sucrose and high fat in these prior studies remain unclear. Therefore, the present study was designed to examine the early effects of high fat or high sucrose feeding on vasodilation in resistance arterioles isolated from mildly overweight animals. Moreover, we examined the potential differential effects of high sucrose vs. high fat diets to elicit changes in endothelial function. We hypothesized that feeding rats diets containing high sucrose or high fat for 6 weeks would produce oxidative stress and inflammation leading to endothelial dysfunction and impaired vasodilation in small mesenteric resistance arterioles. Therefore, the goals of the present study were to *1) *demonstrate that rats fed a high sucrose or high fat diet gain more adiposity compared to Chow fed controls, *2) *show that ROS levels were increased in the plasma as well as in small mesenteric resistance arterioles of high sucrose as well as high fat fed rats, *3) *determine if eNOS protein expression is reduced in rats fed either diet thereby contributing to impaired vasodilation, and *4) *test whether ROS or inflammatory pathways are involved in the impaired endothelium-dependent vasodilation that occurs after 6 weeks of high sucrose or high fat intake.

## Methods

All protocols and surgical procedures employed in this study were reviewed and approved by the Institutional Animal Care and Use Committees of the University of New Mexico School of Medicine and Arizona State University.

### Experimental Groups

Male Sprague-Dawley rats (140-160 g body weight, Harlan Industries) were divided into three groups as shown in table 1. Rats were maintained on the respective diets for 6 weeks and food was replaced every 2-3 days to prevent spoiling. Animals were exposed to a 12:12 h light dark cycle and had free access to food and water. Rats were weighed every week to assess the effect of the diets on weight gain. At the end of each diet, tail vein blood samples were collected after a 12-hour overnight fast to determine plasma triglyceride levels using a CardioCheck PA analyzer with test strips specific for triglycerides (Polymer Technology Services, Indianapolis, IN). Epididymal fat pad mass was determined following the 6-week feeding protocols to assess the effects of the diet on body fat content. This fat pad was chosen since it is well defined and easily extracted thereby minimizing the risk for experimental bias.

**Table 1:** Diet Compositions

*Diet*	*Protein (% kcal)*	*Carbohydrates (% kcal)*	*Fat (% kcal)*	*Total kcal/g*	*Source*
Chow Diet (Chow)	18.9	57.33 (% sucrose NA)	5	3.4	Cat. No. 2018 Harlan Teklad
High Sucrose Diet (HSD)	20	70 (34.5% sucrose)	10	4.73	Cat. No. D12450B Research Diets, Inc
High Fat Diet (HFD)	20	20 (6.8% sucrose)	60	5.24	Cat. No. D12492 Research Diets, Inc

### Oral Glucose Tolerance Tests (OGTT)

At the end of the 6-week feeding protocol, rats were food restricted by providing 4 g/rat of their respective diets at 5 PM the night prior to the OGTT. After an initial tail vein blood draw at 8 AM the following morning (0 min), rats were administered an oral dose of 1 g/kg D-glucose by gavage. Blood samples were taken at 0, 60 and 120 minutes post-glucose administration and whole blood glucose was measured using a glucose meter (CardioChek PA, Polymer Technology Systems, Inc., Indianapolis, IN).

### Measurement of Plasma TBARS

Plasma samples were collected by cardiac puncture from anesthetized rats (sodium pentobarbital, 200 mg/kg i.p.) in all three groups. The level of lipid peroxidation, as a measure of oxidative stress, was analyzed using a thiobarbituric acid reactive substances (TBARS) assay kit from OXItek according to the manufacturer's protocol (Cat No. 0801192, ZeptoMetrix Corp., Buffalo, NY).

### Measurement of Plasma Adipokines

Plasma was obtained from deeply anesthetized rats via cardiac puncture following the 6-week feeding protocol. Plasma was analyzed by radioimmunoassay using a dual antibody technique for rat leptin and adiponectin levels by the Hormone Assay and Analytical Services Core Facility at Vanderbilt University (Nashville, Tennessee).

### Preparation of Experimental Solutions for Isolated Vessel Studies

DCF was dissolved in anhydrous dimethyl sulfoxide (DMSO) at a concentration of 50 μg/ml. Immediately prior to loading, DCF was mixed with a 20% v/v solution of pluronic acid in DMSO, and this mixture was diluted with HEPES buffer to yield a final concentration of 5 μM DCF and 0.05% pluronic acid. Acetylcholine (ACh; 1.0 M; Sigma) and phenylephrine (PE; 1.0 M; Sigma) were dissolved in deionized water, aliquoted, and frozen (-20˚C) until use.

### Isolation of Mesenteric Resistance Arteries

Rats were anesthetized with sodium pentobarbital (200 mg/kg, i.p.) and a midline laparotomy was performed to expose and remove the mesenteric arcade. The arcade was immediately placed in ice-cold HEPES buffer (in mM: 134.4 NaCl, 6 KCl, 1 MgCl_2_, 1.8 CaCl_2_, 10 HEPES, 10 glucose, pH 7.4 with NaOH) pinned out in a Silastic coated dissection dish, and fifth order mesenteric resistance arterioles (~1 mm length; 80-120 μm, i.d.) were isolated. Isolated arterioles were then transferred to a vessel chamber (Living Systems, CH-1) filled with HEPES, cannulated with glass pipettes, and secured in place with silk ligature. The vessels were then stretched longitudinally to approximate *in situ *length, pressurized to 60 mmHg with either a fluid filled column or servo-controlled peristaltic pump (Living Systems Instrumentation, Burlington, VT), and the chamber placed on a microscope stage for analysis (to be described for each procedure). Vessels were superfused with warm (37˚C) physiological salt solution (PSS) containing (in mM): 129.8 NaCl, 5.4 KCl, 0.5 NaH_2_PO_4_, 0.83 MgSO_4_, 19 NaHCO_3_, 1.8 CaCl_2_, and 5.5 glucose at a rate of 10 mL/min. The PSS was aerated with 21% O_2_, 6% CO_2_, balance N_2 _gas mixture throughout the experiments to maintain pH and adequate oxygenation. Viability was verified prior to each experiment by adding the vasoconstrictor phenylephrine (PE; 10^-6 ^M) followed by the endothelium-dependent vasodilator acetylcholine (ACh; 10^-6 ^M) to the superfusate.

### Measurement of Vascular ROS

Vessel chambers were transferred to a Nikon Diaphot 300 microscope equipped with a 10 × fluorescence (FITC) objective for analysis. Following a 30-minute equilibration in aerated PSS, vessels were loaded in the dark with the cell-permeant ROS-sensitive fluorescence indicator, 5-(and-6)-chloromethyl-2',7'-dichlorodihydrofluorescein diacetate, acetyl ester (DCF; Molecular Probes) in a vessel chamber attached to a temperature controller (Living Systems). DCF is oxidized by cytoplasmic peroxynitrite (ONOO^-^), hydrogen peroxide (H_2_O_2_), and hydroxyl radicals (·OH) to produce a fluorescent product [21-23]. Images were collected prior to DCF loading, for background, and 50 minutes later using a cooled, digital CCD camera (SenSys 1400). MetaFluor 4.5 software (Universal Imaging) was used for processing images. Specificity of DCF fluorescence as a measure of ROS was verified in prior experiments in our laboratory [24,25].

### Endothelium-Dependent Vasodilation

After equilibration for 30 min, isolated arteries pressurized at 60 mmHg were superfused for 1 hour with either a control PSS solution or PSS with the addition of antagonists. Vessels were then pre-constricted to 50% of resting inner diameter with PE (Table 2). Vasodilation in response to increasing concentrations of ACh (10^-9 ^to 10^-5 ^M, 3 min each step) in the superfusate was then determined by measuring the intraluminal diameter (i.d.) using a video dimension analyzer (Living Systems, Burlington, VT). Data were digitized using DATAQ A/D software for analysis (DATAQ Instruments, Akron, OH). Additional experiments measuring the effectiveness of the SOD mimetic, 4,5-Dihydroxy-1,3-benzene-disulfonic acid (tiron; 10 mM), and catalase (1200 U/mL) at restoring ACh-induced vasodilation were performed. Responses to ACh in the presence of the synthetic manganese-porphyrin complex, chloro[[2,2'-[1,2-ethanediyl*bis*[(nitrilo-κN)methylidyne]]*bis*[6-methoxyphenolato-κO]]]-manganese (EUK-134; 10 μM; Cayman Chemical Company, Ann Arbor, MI), were also examined. This compound is an SOD mimetic with enhanced catalase activity [26]. Inhibition of nitric oxide synthase, using 100 μM Nω-nitro-L-arginine (LNNA; Sigma), was performed to determine the role of NO in ACh-induced vasodilation. Further studies to assess the combined effects of LNNA and tiron and catalase or EUK-134 on vasodilatory responses were performed. Vascular reactivity in the presence of the hydrogen peroxide scavenger catalase (1200 U/mL) or an inhibitor of cyclooxygenase (COX) (indomethacin, 10 μM) was also examined. Vessels were subsequently superfused for 30 min with calcium-free PSS (in mM/L: 129.8 NaCl, 5.4 KCl, 0.5 NaH_2_PO_4_, 0.83 MgSO_4_, 19.0 NaHCO_3_, 5.5 glucose, and 3.0 EGTA) to obtain the passive inner diameter from which total constriction and subsequent percent vasodilation were calculated.

**Table 2:** Physical and Biochemical Parameters

*Parameter*	*Chow*	*HSD*	*HFD*	*n*
**Initial Body Weight (g)**	151.5 ± 1.68	151.3 ± 1.35	150.6 ± 1.58	*44-47*
**Final Body Weight (g)**	336.6 ± 3.63	359.7 ± 3.84*	371.3 ± 4.00*^#^	*44-47*
**6 Week Weight Gain (g)**	185.1 ± 3.62	208.5 ± 4.10*	220.8 ± 4.37*^#^	*44-47*
**Epididymal Fat Pad Mass (g)**	2.72 ± 0.15	4.00 ± 0.27*	5.61 ± 0.38*^#^	*9-10*
**Plasma Triglycerides (mg/dL)**	51.50 ± 0.92	95.00 ± 8.11*	52.11 ± 2.11^#^	*9-10*
**Plasma Leptin (ng/mL)**	1.18 ± 0.25	2.15 ± 0.36*	3.50 ± 0.54*	*5-7*
**Plasma Adiponectin (μg/mL)**	18.17 ± 2.41	16.31 ± 2.77	17.32 ± 1.27	*5-13*
**Plasma TBARS (nM/L)**	21.29 ± 2.20	32.54 ± 2.56*	29.88 ± 1.59*	*9-10*

Data expressed as means ± SEM; *p < 0.05 from Chow; #*p *< 0.05 from HSD.

### Vascular Smooth Muscle Sensitivity to NO

After equilibration for 30 min, isolated arteries pressurized at 60 mmHg were superfused for 1 hour with PSS. Vessels were then pre-constricted to 50% of resting inner diameter with increasing concentrations of PE. Vasodilation in response to the NO donor sodium nitroprusside (10^-11 ^to 10^-3 ^M, 3 min each step) in the superfusate was then determined by measuring the intraluminal diameter (i.d.) using a video dimension analyzer (Living Systems, Burlington, VT). Vessels were subsequently superfused for 30 min with calcium-free PSS (in mM/L: 129.8 NaCl, 5.4 KCl, 0.5 NaH_2_PO_4_, 0.83 MgSO_4_, 19.0 NaHCO_3_, 5.5 glucose, and 3.0 EGTA) to obtain the passive inner diameter from which total constriction and subsequent percent vasodilation were calculated. Data were digitized using DATAQ A/D software for analysis (DATAQ Instruments, Akron, OH).

### Western Blots for Endothelial Nitric Oxide Synthase (eNOS)

Mesenteric arteries were isolated and snap-frozen in liquid nitrogen. Frozen arteries were homogenized in ice-cold Tris-HCl homogenization buffer containing 10 mM Tris (pH 7.6), 1 mM EDTA, 1% triton X-100, 0.1% Na-deoxycholate, 0.03% protease inhibitor cocktail (Sigma P2714), and 1 mM phenylmethanesulfonyl fluoride (PMSF) using a ground glass homogenizer. Homogenates were then spun at 4000 g for 10 min at 4˚C to remove insoluble debris. Protein concentration in the supernatant was analyzed using the Bradford method (Bio-Rad, Hercules, CA). Tissue sample proteins (25 μg/lane) were resolved by 7.5% Tris-HCl sodium dodecyl sulfate polyacrylamide gel electrophoresis (SDS-PAGE) (Bio-Rad, Hercules, CA). The separated proteins were then transferred to Immuno-Blot polyvinylidene difluoride (PVDF) membranes (Bio-Rad, Hercules, CA). The membranes were subsequently incubated in blocking buffer (100 ml Tween/Tris-buffered saline (TTBS), 3% BSA, 5% nonfat dry milk) for 1 hr at room temperature. Following washes in TTBS, PVDF membranes were incubated overnight at 4˚C with mouse monoclonal antibody specific for eNOS (1:2500; Cat. 610296; BD Transduction Laboratories, San Jose, CA) and rabbit polyclonal antibody to β-actin as a loading control (1:10,000; Cat. Ab8227; AbCam, Cambridge, MA). Membranes were then washed in TTBS and incubated with anti-mouse and anti-rabbit horseradish peroxidase-conjugated secondary antibodies (1:5000, Cat. PI-2000 and PI-1000; Vector Laboratories, Burlingame, CA) for 1 hr at room temperature followed by washes in Tris-buffered saline (TBS) and a 1 min exposure to Pierce enhanced chemiluminescence western blotting substrate (Thermo Scientific, Rockford, IL). Immunoreactive bands were visualized by exposure to x-ray film (Kodak X-OMAT, Thermo Fisher Scientific, Pittsburgh, PA). The developed films were analyzed using ImageJ software (NIH) and eNOS protein levels were normalized to β-actin.

### Statistics

Data are expressed as means ± SEM. Analysis of variance (ANOVA) was used to compare physical and biochemical data (Table 2) as well as eNOS protein expression data within and between groups. Data from the oral glucose tolerance test were analyzed using repeated measures ANOVA. Percent vasodilation to ACh and SNP were calculated as the percent reversal of tone elicited by phenylephrine as well as inherent myogenicity. This is determined by measuring the difference in intraluminal diameter observed at each concentration of ACh or SNP vs. the inner diameter observed in a calcium free solution designed to eliminate all tone. Percentage data were arcsine transformed to approximate normal distribution prior to analysis by two-way repeated measures ANOVA. Where significant effects were indicated by ANOVA, individual groups were compared using Student-Newman-Keuls post hoc analysis. A probability of ≤ 0.05 was accepted as statistically significant for all comparisons.

## Results

### Diet

Both HSD and HFD rats gained significantly more weight and epididymal fat pad mass over the course of the 6-week feeding protocol compared to Chow fed rats, with the HFD rats gaining the most (Table 2). This represents an overall weight gain that was 11.2% greater than Chows in the HSD group and 16.2% greater in the HFD group. Despite the significant increases in weight by rats in each experimental feeding protocol, according to Lee's Index of obesity ((1/3(body weight(g)/nasoanal length (mm)) × 10000); data not shown) it was not sufficient to classify the animals as obese, but rather, a mild increase in body weight was developed.

### Plasma Triglycerides and Whole Blood Glucose Measurements

**Figure 1:**
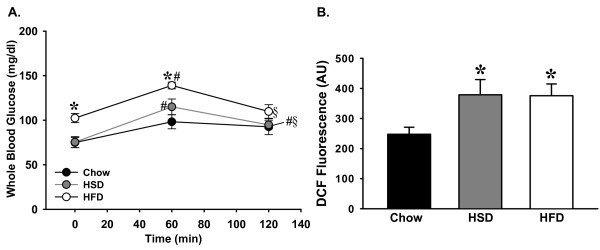
**Panel A: Whole blood glucose levels during an oral glucose tolerance test in Chow (*n *= 5-7), HSD (*n *= 5), and HFD (*n *= 5) fed rats**. Rats were food restricted by providing 4 g/rat of their respective diets at 5 PM the night prior to the OGTT. At 8 am the following morning, rats were administered an oral bolus of glucose (1 g/kg D-glucose) by gavage immediately following the initial glucose sample (0 min). Glucose levels returned to baseline by 120 mins in the Chow and HFD fed rats but remained elevated in HSD fed rats. Data expressed as means ± SEM. *p < 0.05 vs. corresponding time point in Chows; #*p *< 0.05 vs. initial blood glucose level (time 0); §*p *< 0.05 vs. corresponding level at 60 mins. **Panel B**: Measurement of oxidative stress in isolated mesenteric resistance arterioles from rats following the 6-week feeding protocol. 5-(and-6)-chloromethyl-2',7'-dichlorodihydrofluorescein diacetate, acetyl ester (DCF) fluorescence (average gray scale values above background) in isolated mesenteric resistance arteries from Chow (*n *= 6), HSD (*n *= 5) and HFD (*n *= 5) fed rats. Data are expressed as means ± SEM. *p ≤ 0.05 vs. Chow fed controls

Plasma triglyceride levels were significantly elevated in the HSD group compared to Chow and HFD rats (Table 2). Fasting glucose levels were significantly elevated in the HFD fed rats (Figure 1A). Sixty minutes after an oral glucose dose, plasma glucose levels remained above baseline in both HFD and HSD fed rats but returned to fasting levels in the Chow fed controls. By 120 minutes, glucose levels in the HFD fed rats returned to fasting levels and were not significantly different from Chows, whereas levels in HSD rats remained significantly elevated from initial baseline glucose concentrations.

### Levels of Circulating Adipokines

Plasma leptin levels, which are positively correlated with adiposity, were elevated in both HSD and HFD rats (Table 2). There were no significant differences in plasma adiponectin levels between the three groups of rats (Table 2).

### Measurement of Plasma and Vascular ROS

Plasma TBARS, a measure of whole animal oxidative stress, were significantly elevated in both the HSD and HFD fed rats compared to Chow fed controls (Table 2). Levels of ROS (ONOOˉ, ˙OH, and H_2_O_2_) assessed by DCF fluorescence were also significantly elevated in mesenteric resistance arterioles isolated from both HSD and HFD fed rats compared to Chow fed controls (Figure 1B).

### ROS-Mediated Vascular Dysfunction

**Figure 2:**
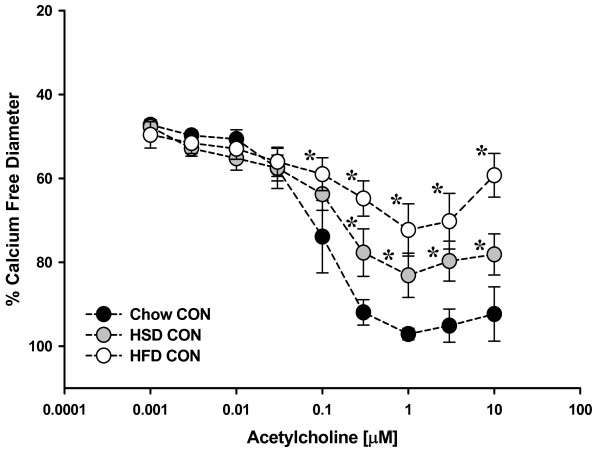
**Endothelium-dependent vasodilation in isolated small mesenteric arterioles from rats fed either Chow, HSD, or HFD (*n *= 6-8 per group)**. Vasodilatory responses to acetylcholine were measured in pre-constricted arteries (50% of resting inner diameter). There were no significant differences in pre-constricted diameters between, or within, groups (Table 2). Compared to Chow fed controls, arteries from HSD and HFD rats exhibited significantly attenuated vasodilation. Data are expressed as means ± SEM. *p ≤ 0.05 vs. Chow controls.

**Figure 3:**
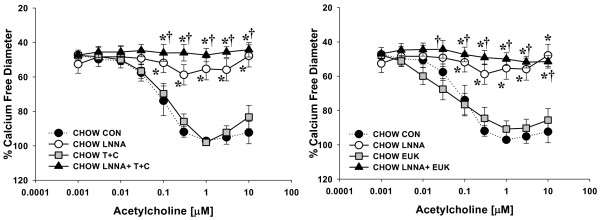
**Acetylcholine-mediated vasodilatory responses in isolated mesenteric resistance arteries from Chow fed rats**. Vasodilatory responses to acetylcholine (ACh) were measured in the presence and absence of the NOS inhibitor Nω-nitro-L-arginine (LNNA; 100 μM) in small mesenteric arteries (*n *= 6-8). Arteries were exposed to LNNA in the lumen and superfusate prior to pre-constriction with phenylephrine to 45-50% resting inner diameter. Vasodilatory responses to acetylcholine (ACh) were also measured in the presence and absence of antioxidants (*n *= 4-5). For these studies, arteries were pre-constricted as previously described. Treated arteries were pre-exposed to the ROS scavengers 4,5-Dihydroxy-1,3-benzene-disulfonic acid (tiron; 10 mM) and catalase (1200 U/mL) or EUK-134 (10 μM). Separate arteries were exposed to the nitric oxide synthase inhibitor LNNA (100 μM) or LNNA with the addition of EUK-134 or tiron and catalase. The Chow control data (dotted line) are repeated from figure 2 for comparison. Data are expressed as means ± SEM. *p ≤ 0.05 vs. controls; **†***p *< 0.05 vs. EUK-134 or T+C only.

**Figure 4:**
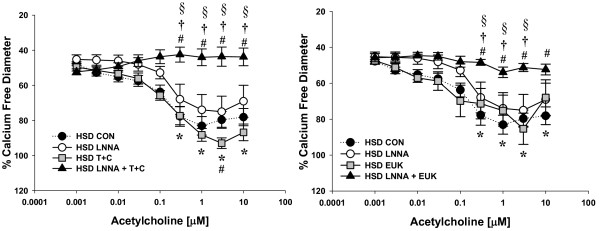
**Acetylcholine-mediated vasodilatory responses in isolated mesenteric resistance arteries from HSD fed rats**. Vasodilatory responses to acetylcholine (ACh) were measured in the presence and absence of the NOS inhibitor Nω-nitro-L-arginine (LNNA; 100 μM) in small mesenteric arteries (*n *= 6-8). Arteries were exposed to LNNA in the lumen and superfusate prior to pre-constriction with phenylephrine to 45-50% resting inner diameter. Vasodilatory responses to acetylcholine (ACh) were also measured in the presence and absence of antioxidants HSD (*n *= 4-9). For these studies, arteries were pre-exposed to the ROS scavengers 4,5-Dihydroxy-1,3-benzene-disulfonic acid (tiron; 10 mM) and catalase (1200 U/mL) or EUK-134 (10 μM). Separate arteries were exposed to the nitric oxide synthase inhibitor LNNA (100 μM) or LNNA with the addition of EUK-134 or tiron and catalase. The HSD control data (dotted line) are repeated from figure 2 for comparison.Data are expressed as means ± SEM. *p ≤ 0.05 HSD controls vs. Chow controls; #*p *≤ 0.05 vs. HSD controls; **†***p *< 0.05 vs. HSD EUK-134 or T+C only; §*p *≤ 0.05 vs. HSD LNNA only.

**Figure 5:**
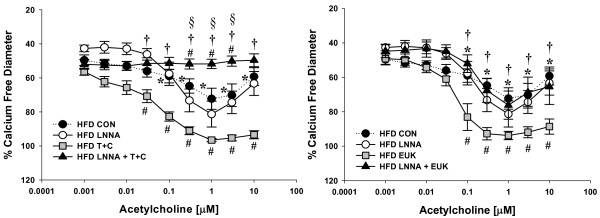
**Acetylcholine-mediated vasodilatory responses in isolated mesenteric resistance arteries from HFD fed rats**. Vasodilatory responses to acetylcholine (ACh) were measured in the presence and absence of the NOS inhibitor Nω-nitro-L-arginine (LNNA; 100 μM) in small mesenteric arteries (*n *= 6-8). Arteries were exposed to LNNA in the lumen and superfusate prior to pre-constriction with phenylephrine to 45-50% resting inner diameter. Vasodilatory responses to acetylcholine (ACh) were also measured in the presence and absence of antioxidants (*n *= 5-8). For these studies, arteries were pre-exposed to the ROS scavengers 4,5-Dihydroxy-1,3-benzene-disulfonic acid (tiron; 10 mM) and catalase (1200 U/mL) or EUK-134 (10 μM). Separate arteries were exposed to the nitric oxide synthase inhibitor LNNA (100 μM) or LNNA with the addition of EUK-134 or tiron and catalase. The HFD control data (dotted line) are repeated from figure 2 for comparison. Data are expressed as means ± SEM. *p ≤ 0.05 HFD controls vs. Chow controls; #*p *≤ 0.05 vs. HFD controls; **†***p *< 0.05 combined HFD LNNA + EUK-134 or T+C vs. HFD EUK-134 or T+C only; §*p *≤ 0.05 combined HFD LNNA T+C vs. HFD LNNA only.

**Figure 6:**
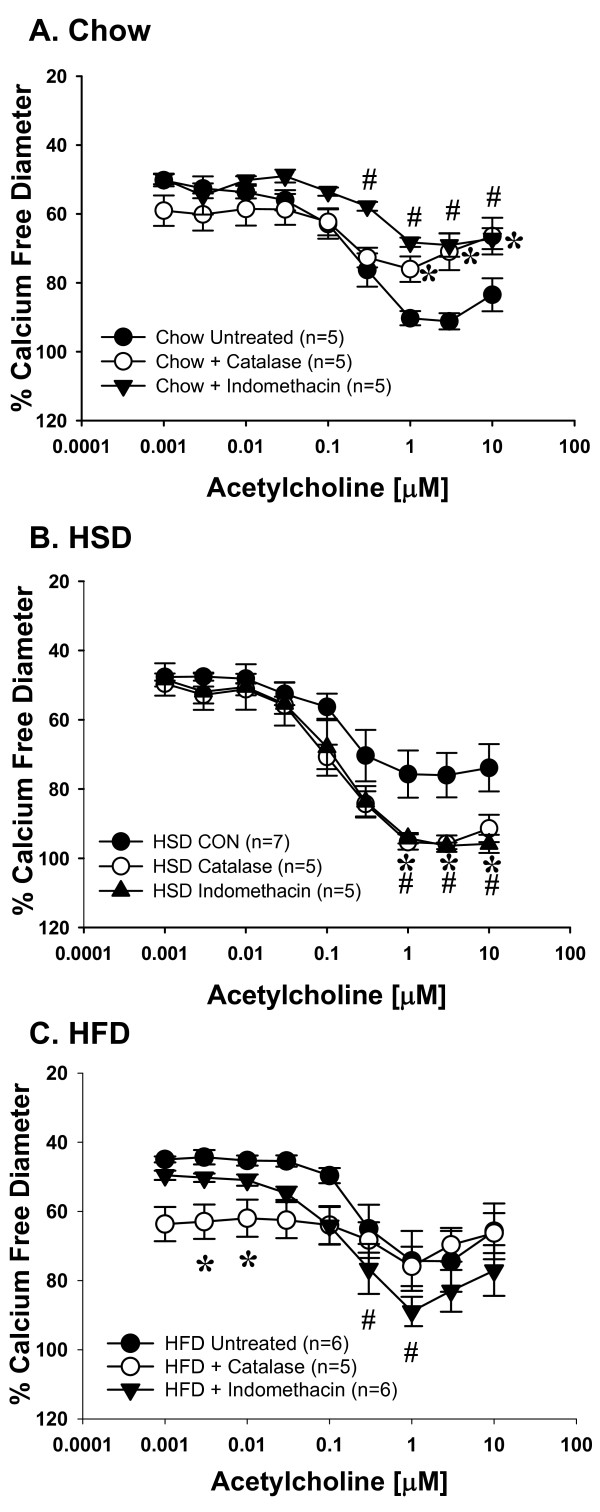
**Acetylcholine-mediated vasodilatory responses in isolated mesenteric resistance arteries following inhibition of COX (100 μM indomethacin) or hydrogen peroxide (1200 U/mL catalase)**. **Panel A**: Data from Chow-fed rats. **Panel B**: Data from HSD-fed rats. **Panel C**: Data from HFD-fed rats. All data expressed as means ± SEM. *p < 0.05 catalase vs. respective untreated control (CON); #*p *< 0.05 indomethacin vs. respective untreated control (CON).

ACh-induced vasodilation was significantly impaired in mesenteric resistance arterioles from both HSD and HFD fed rats compared to Chow fed controls (Figure 2). Incubation with the NOS inhibitor, LNNA (100 μM) had no significant effect on ACh-mediated vasodilation in arteries from HSD and HFD fed rats whereas Chow fed rats demonstrated significant attenuation of relaxation (Figures 3, 4, 5). These data demonstrate that ACh-mediated vasodilation is impaired following high calorie feeding and that this impairment is associated with a reduced vasodilatory role of NO. The addition of tiron (10 mM) and catalase (1200 U/mL) or EUK-134 (10 μM) had no significant effect on ACh-induced vasodilation in arteries from Chow fed control rats (Figure 3). In contrast, tiron and catalase were effective at normalizing vasodilatory responses in vessels from both HSD and HFD fed rats (Figures 4 &5), whereas EUK-134 only restored vasodilation in vessels from HFD fed rats (Figure 5). There were no significant differences in vasodilation responses in untreated and EUK-134 treated vessels from HSD rats (Figure 4). The combined effects of LNNA and EUK-134 or tiron and catalase were also assessed. The combined incubation of arteries from Chow fed rats did not result in further attenuation of vasodilation compared to LNNA treatment alone (Figure 3). In contrast, the combined treatment of arteries from HSD and HFD rats with LNNA and tiron and catalase resulted in significantly blunted vasodilation (Figures 4 &5). Incubation of arteries with LNNA and EUK-134 from HSD rats also significantly blunted vasodilation (Figure 4), but produced no further impairment of vasodilation in arteries from HFD rats compared to untreated HFD arteries (Figure 5). Incubation of arteries with the H_2_O_2 _scavenger catalase significantly impaired vasodilation in arteries from Chow rats supporting a normal vasodilatory role of H_2_O_2 _in this group (Figure 6A). In contrast, incubation of arteries from HSD rats with catalase normalized ACh-mediated vasodilation but did not improve vasodilation of arteries from HFD rats (Figures 6B &6C). Inhibition of COX using indomethacin significantly impaired vasodilation in arteries from Chow rats, but completely normalized vasodilation in arteries from HSD rats and mildly improved vasodilation in arteries from HFD rats (Figures 6A-C).

### Vascular Smooth Muscle Sensitivity to Nitric Oxide (NO)

**Figure 7:**
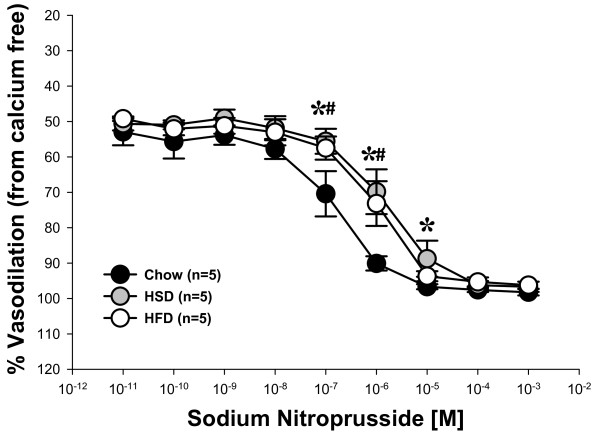
**Vasodilatory responses to the NO donor sodium nitroprusside in isolated mesenteric arteries from Chow, HSD and HFD rats**. Data expressed as means ± SEM. *p ≤ 0.05 Chow vs. HSD; #*p *≤ 0.05 Chow vs. HFD.

Compared to Chow fed rats, those fed either HSD or HFD developed significantly impaired vascular smooth muscle sensitivity to NO as indicated by blunted responses to the NO donor sodium nitroprusside (Figure 7).

### Western Blots for Endothelial Nitric Oxide Synthase (eNOS)

**Figure 8:**
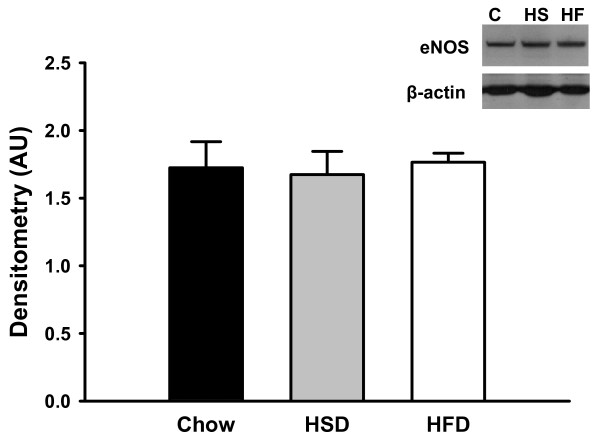
**Immunostaining of endothelial nitric oxide synthase (eNOS) in mesenteric artery homogenates from Chow (C), HSD (HS), and HFD (HF) fed rats**. Densitized values of the immunoblot were normalized to β-actin protein concentration and are expressed as a ratio (mean ± SEM). Inset: representative blot of eNOS and β-actin in isolated rat mesenteric arteries from rats in all three treatment groups. *n *= 5-6 per group. Data are not significantly different.

Protein concentration of eNOS in mesenteric arteries was not significantly different between groups (Figure 8).

## Discussion

The major findings of this study are that 6 weeks of high sucrose or high fat feeding results in increased body mass and adiposity (Table 2) concomitant with increased oxidative stress and impaired vasodilation, although the mechanism of impaired vasodilation differed for each group. Vasodilation of arteries from Chow fed rats is dependent on NO as well as H_2_O_2 _(Figures 3 and 6). Feeding rats either HSD or HFD results in diminished vascular smooth muscle sensitivity to NO (Figure 7). Vasodilation is further impaired in rats fed HSD as a result of increases in H_2_O_2 _which acts to oppose vasodilation (Figures 1, 4 &6). In contrast, the findings of this study implicate O_2_˙ˉ-mediated scavenging of NO to form ONOO- in arteries from rats fed HFD (Figures 1 &5).

Feeding rats a HSD diet alone elicited hypertriglyceridemia (Table 2), impaired glucose tolerance (Figure 1A), hyperleptinemia (Table 2), enhanced oxidative stress (Table 2 & Figure 1B) and impaired acetylcholine-mediated vasodilation (Figure 2), which are characteristics of metabolic syndrome and pre-diabetes. Animals on the HFD gained more adiposity compared to the HSD rats (Table 2) and demonstrated further characteristics of metabolic syndrome as they developed fasting hyperglycemia (Figure 1A) in addition to hyperleptinemia (Table 2), oxidative stress (Table 2 & Figure 1B), and impaired acetylcholine-induced vasodilation (Figure 2). These significant alterations in oxidative stress and vascular reactivity demonstrate that vascular dysfunction occurs well before the development of obesity and that different diets can have varying effects on biochemical parameters and vascular reactivity in rats.

Elevated plasma glucose, free fatty acids and triglycerides contribute to increased ROS production [9-11]. Previous studies have shown that feeding obesity-prone Sprague-Dawley rats a moderately high fat diet (32% kcal% as fat) for 16 weeks results in elevated TBARS and O_2_˙ˉ in aorta [27]. Similarly, our studies demonstrate increased fasting glucose in the HFD rats (Figure 1A) and plasma triglycerides in the HSD rats (Table 2) with both groups of rats developing elevated TBARS and DCF fluorescence, indicative of vascular ROS (Table 2 & Figure 1B).

Multiple oxidative stress pathways can potentially lead to decreases in NO bioavailability and therefore, a reduction in vasodilatory responses. A direct pathway occurs by O_2_˙ˉ-induced scavenging of endothelial-derived NO to form peroxynitrite (ONOO-) [6,7]. Another mechanism involves the uncoupling of eNOS as a result of diminished levels of the essential eNOS cofactor, tetrahydrobiopterin, resulting in enhanced O_2_˙ˉ formation and further decreases in NO levels [6-8,11]. Furthermore, ONOO- itself can oxidize and deactivate tetrahydrobiopterin [28]. Moreover, H_2_O_2_, produced from the breakdown of O_2_˙ˉ, is reported to exhibit vasodilatory or vasoconstrictor effects depending on the concentration or tissue examined [29-31]. Since the observed increase in oxidative stress in HSD and HFD rats may directly affect blood vessel reactivity or impact NO bioavailability, the effects of oxidative stress on endothelium-mediated vasodilation were measured (Figures 3, 4, 5, 6).

Data from the present study demonstrate that high sucrose and high fat feeding result in significantly attenuated endothelium-dependent vasodilation compared to Chow-fed controls (Figure 2). In Chow-fed rats, ACh-mediated vasodilation appears to rely on NO since the NOS inhibitor LNNA nearly abolished the vasodilatory response (Figure 3). In contrast, inhibition of NOS caused no further impairment of vasodilation in HSD and HFD fed rats (Figures 4 &5). These data suggest that ROS may be involved in the scavenging of NO in these animals as eNOS protein expression levels were not significantly different in the experimental groups compared to Chow fed rats (Figure 8). Further studies demonstrated that arteries from HSD and HFD rats have impaired vascular smooth muscle sensitivity to NO contributing to the impaired ACh-mediated vasodilation (Figure 7). Therefore, it is evident that the residual response to ACh following high calorie feeding is NO-independent and likely involves other endothelium-dependent vasodilatory pathways.

Since the observed increase in oxidative stress in HSD and HFD rats may impact NO bioavailability, the effects of oxidative stress on endothelium-mediated vasodilation were measured (Figures 3, 4, 5, 6). ACh-mediated vasodilation responses in arteries from HFD rats were greatly attenuated across a broad range of ACh doses and were normalized by the inhibition of ROS using either tiron and catalase or EUK-134 (Figure 5). In contrast, vasodilation of arteries from HSD rats was impaired at only high doses of ACh which likewise demonstrated improved vasodilation in the presence of tiron and catalase but not EUK-134 (Figure 4). These data illustrate that oxidative stress plays a role in the limitation of ACh-mediated vasodilation in HSD and HFD rats with only modest increases in adiposity. Oxidative stress is similarly involved in the impaired vasodilatory responses of aortic rings and renal arterioles from alloxan-induced diabetic rabbits [32]. Impaired endothelium-dependent vasodilation, as occurs in coronary artery diseases, has likewise been linked with increased oxidative stress since administration of the antioxidant vitamin C improved the response in humans [33]. Similarly, Zucker obese rats exhibit attenuated responses to ACh in isolated coronary as well as middle cerebral arteries [34,35]. Moreover, diminished ACh-mediated vasorelaxation in thoracic aorta from insulin resistant rats has been observed [36].

To test whether impaired vasodilation in arteries from HSD and HFD is due to an interaction of ROS and NO, vessels from each group were incubated in the presence of both the NOS inhibitor LNNA and the ROS scavengers tiron and catalase or EUK-134. Data from these studies demonstrate that impaired vasodilatory responses recorded in arteries from HSD rats is at least in part mediated by reduced NO bioavailability as the combined treatment nearly abolished vasodilation (Figure 4). Similar results were observed in arteries from HFD rats treated with LNNA and tiron and catalase, suggesting that ROS may also reduce the bioavailability of NO in these vessels (Figure 5). However, superfusion of arteries with LNNA and EUK-134 did not normalize vasodilation in arteries from these rats (Figure 5). This apparent discrepancy may be due to the different mechanisms of action of each of these ROS scavengers.

Since H_2_O_2 _is known to exert both vasodilatory and vasoconstrictor properties, we examined the role of H_2_O_2 _by exposing arteries to catalase in the absence of superoxide dismutase mimetics. In arteries from HFD rats, this exposure did not improve vasodilation (Figure 6C) whereas the combined superoxide dismutase mimetics and catalase were successful at normalizing vasodilation (Figure 5). This suggests that in HFD vessels, elevated O_2_˙ˉ may be responsible for scavenging of NO resulting in the production of ONOOˉ. In contrast, arteries from HSD fed rats demonstrate only a mild improvement in ACh-mediated vasodilation in the presence of tiron and catalase but no effect of EUK-134 (Figure 4). Since inhibition of H_2_O_2 _alone normalized vasodilation in this group (Figure 6B), this supports a role for H_2_O_2 _as a vasoconstrictor following HSD feeding. DCF can be oxidized by both ONOOˉ and H_2_O_2 _resulting in increased fluorescence as we observed in arteries from HSD and HFD rats (Figure 1B). Our data also demonstrate a physiological role of H_2_O_2 _in vasodilatory responses of arteries from Chow rats (Figure 6A), that has been described by others [29-31].

We also observed a differential role of COX products between the different feeding regimens. In Chow rats, COX inhibition blunted ACh-mediated vasodilation (Figure 6A). In contrast, indomethacin normalized vasodilation in arteries from HSD rats suggesting a switch to vasoconstrictor COX products following high sucrose feeding (Figure 6B) that was only minimally present in the HFD group (Figure 6C). Thus, the profile of COX-derived vasoactive products may be altered by diet.

In summary, our data demonstrate that feeding rats either a high fat or high sucrose diet results in the development of oxidative stress as well as impaired vasodilation. The data highlight the importance of the type of diet as it can produce divergent effects on vascular reactivity pathways despite both groups developing increased body mass, adiposity and oxidative stress. Although rats in the HSD fed group develop similar levels of oxidative stress as observed in the HFD rats, the impaired vasodilation is not as severe and the mechanisms of impaired vasodilation are divergent. In the HFD group, the impaired vasodilation appears to be mediated in part by O_2_˙ˉ scavenging of NO. In contrast, H_2_O_2 _is implicated in the impaired vasodilatory responses in vessels from HSD rats. In conclusion, the impaired vasodilatory responses to acetylcholine in rats fed either HSD or HFD are mediated by ROS scavenging of NO, impaired smooth muscle sensitivity to NO as well as by inflammatory factors.

## List of abbreviations used

ANOVA: Analysis of Variance; ACh: acetylcholine; DCF: 5-(and-6)-chloromethyl-2',7'-dichlorodihydrofluorescein diacetate, acetyl ester; DMSO: dimethyl sulfoxide; eNOS: endothelial nitric oxide synthase; EUK-134: chloro[[2,2'-[1,2-ethanediyl*bis*[(nitrilo-κN)methylidyne]]*bis*[6-methoxyphenolato-κO]]]-manganese; H_2_O_2_: hydrogen peroxide; HFD: high fat diet; HSD: high sucrose diet; LNNA: Nω-nitro-L-arginine; NO: nitric oxide; O_2_˙ˉ, superoxide; OGTT: oral glucose tolerance test; ˙OH: hydroxyl radical; ONOOˉ: peroxynitrite; PE: phenylephrine; PMSF: phenylmethanesulfonyl fluoride; PSS: physiological salt solution; PVDF: polyvinylidene difluoride; ROS: reactive oxygen species; SDS-PAGE: sodium dodecyl sulfate polyacrylamide gel electrophoresis; SNP: sodium nitroprusside; SOD: superoxide dismutase, TBARS: thiobarbituric acid reactive substances; tiron, 4,5-Dihydroxy-1,3-benzene-disulfonic acid; TBS: Tris-buffered saline; TTBS: Tween/Tris-buffered saline.

## Competing interests

The authors declare that they have no competing interests.

## Authors' contributions

KLS and BRW conceptualized and designed the study. KLS performed all experiments, assays (with the exception of the leptin and adiponectin assays which were conducted by the Hormone Assay and Analytical Services Core Facility at Vanderbilt University), statistical analyses and wrote the first draft of the manuscript. ML assisted with the experiments and analyses. BRW contributed to the writing of the manuscript, data presentation, interpretation and analyses. All authors read and approved the final manuscript.
